# Omics Studies Revealed the Factors Involved in the Formation of Colony Boundary in *Myxococcus xanthus*

**DOI:** 10.3390/cells8060530

**Published:** 2019-06-03

**Authors:** Mian Nabeel Anwar, Zhi Feng Li, Ya Gong, Raghvendra Pratap Singh, Yue-Zhong Li

**Affiliations:** 1 State Key Laboratory of Microbial Technology, Institute of Microbial Technology, Shandong University, Qingdao 266237, China; miannabeelanwar@gmail.com (M.N.A.); lizhifeng@sdu.edu.cn (Z.F.L.); gongya_seven@163.com (Y.G.); singh.dr.raghvendra@gmail.com (R.P.S.); 2 Department of Research and Development, Uttaranchal University, Dehradun 248007, India

**Keywords:** type VI secretion system, proteomics, *Myxococcus xanthus*, secondary metabolites, microscopy, cold shock proteins

## Abstract

Two unrecognizable strains of the same bacterial species form a distinct colony boundary. During growth as colonies, *Myxococcus xanthus* uses multiple factors to establish cooperation between recognized strains and prevent interactions with unrecognized strains of the same species. Here, *ΔMXAN_0049* is a mutant strain deficient in immunity for the paired nuclease gene, *MXAN_0050*, that has a function in the colony-merger incompatibility of *Myxococcus xanthus* DK1622. With the aim to investigate the factors involved in boundary formation, a proteome and metabolome study was employed. Visualization of the boundary between DK1622 and Δ*MXAN_0049* was done scanning electron microscope (SEM), which displayed the presence of many damaged cells in the boundary. Proteome analysis of the DK1622- boundary disclosed many possible proteins, such as cold shock proteins, cell shape-determining protein MreC, along with a few pathways, such as RNA degradation, phenylalanine, tyrosine and tryptophan biosynthesis, and Type VI secretion system (T6SS), which may play major roles in the boundary formation. Metabolomics studies revealed various secondary metabolites that were significantly produced during boundary formation. Overall, the results concluded that multiple factors participated in the boundary formation in *M. xanthus*, leading to cellular damage that is helpful in solving the mystery of the boundary formation mechanism.

## 1. Introduction

Close relatives of some bacterial species, such as *Proteus mirabilis*, *Bacillus subtilis*, and *Myxococcus xanthus*, are able to form visible boundaries to separate the swarming colonies of different strains that are unrecognizable from each other [1,2,3,4,5]. The phenomenon is known as colony-merger incompatibility [1]. A mechanism for the colony-merger incompatibility is that a donor cell transports effector proteins to a recipient cell in close proximity through the type VI secretion system (T6SS) apparatus in a contact-dependent process, and myxobacteria use the contact-dependent mechanism for outer membrane exchange (OME) [6,7,8,9,10,11,12]. It was also reported that formation of the boundary is due to the delivery of toxins by OME [13]. However, what happens in the boundary and how the boundaries form between the incompatible cells remains unclear.

Recently, proteomic and metabolomic techniques for bacterial studies have become cutting-edge. Proteomics correlate the overall functions of cellular protein compositions and their activities [14]. On the other hand, the utilization of metabolite profiling studies helps to identify, categorize, and analyze chemical compounds [15,16]. Due to various stress conditions, the protein and the metabolic components within a bacterial cell differ from cell to cell. Hence, proteome analyses along with metabolomic studies are able to reveal even minor changes in proteins or metabolites, which is vital to understanding their cellular functions, biological pathways, and different interaction mechanisms [16,17,18,19].

The *M. xanthus* is a Gram-negative soil bacterium that is able to display multicellular behavior along with nascent variation according to its surrounding environment [20]. Previously, we made insertion mutations in the model strain, *M. xanthus* DK1622, to screen mutants deficient in colony-merger compatibility, and 11 mutants were found incompatible with the ancestral strain and formed boundaries [21]. We determined that the *MXAN_0050* and *MXAN_0049* gene pair, which encodes a nuclease toxin and an immunity protein system, is involved in the colony-merger incompatibility of *M. xanthus* DK1622 [10]. The deletion of *MXAN_0049*, a gene encoding an immunity protein for the nuclease toxin encoded by *MXAN_0050*, which is delivered via T6SS, makes the mutant deficient in colony discrimination. In this study, we investigated the morphologies of the colony boundaries between *M. xanthus* DK1622 and the incompatible mutant, Δ*MXAN_0049*, to gain more insight into the cell status within the boundary by staining technique, light, and SEM. We determined the potential factors for the formation of colony boundaries using proteomics. Moreover, the presence of different secondary metabolites was detected by high performance liquid chromatography tandem mass spectrometry (HPLC-MS-MS). Our results revealed the factors participated in cellular damage that lead to the boundary formation in *M. xanthus*.

## 2. Materials and Methods

### 2.1. Bacterial Strains and Culture Conditions

*M. xanthus* DK1622 and its mutants were cultivated at 30 °C in liquid casitone tris medium (CTT) or on CTT plates supplemented with 1.5% agar [22].

### 2.2. Fluorescence Microscopy Observation of Colony Boundaries

Boundaries were observed on CTT plates under an SMZ100 dissection microscope. The boundary cell status was determined by using LIVE/DEAD^®^ *Bac*Light™ Bacterial Viability Kits, L7012 (Invitrogen, Carlsbad, CA, USA), as previously mentioned [21].

### 2.3. Scanning Electron Microscopy (SEM)

To check the condition of cells within the boundary in detail, we used Quanta 250 FEG-USA FEI-SEM (Thermo Fisher Scientific Electron Microscopy, Hillsboro, OR, USA). For sample preparation, we followed the standard protocol with some modifications [23]. Firstly, we cut the area of interaction along with agar. The samples were fixed overnight at 4 °C in a 2.5% glutaraldehyde solution (100 mL of concentrated sample solution, 0.9 mL of 25% glutaraldehyde solution) in a sterile petri plate, in which the surfaces of the samples were covered with fixation solution. After overnight incubation, the fixation solution was removed carefully, and the samples were rinsed once with 0.1 M, pH 7.0 phosphate buffer for 15 min. Later, the samples were subjected to dehydration treatment with concentration gradients (30%, 50%, 70%, 90%, and 95%) of ethanol solution, treated at each concentration for 15–20 min, and, after adding 100% ethanol for the last time, were placed for five hours. After dehydration treatment, the ethanol was discarded, and the samples were cut into a quarter and covered by a glass surface coated with a layer of silver glue. The samples were kept in the critical point drying machine EM CPD300 drying system (Leica, Vienna, Austria) for 45 min to absorb the moisture from the sample and were then coated with gold film. At last, the samples were placed in SEM for observation.

### 2.4. Sample Preparation for LC-MS/MS

Cells were collected for protein extraction by taking only the boundary portion (DK1622-Δ*MXAN_0049*), while the control was collected where DK1622-DK1622 and *ΔMXAN_0049*-*ΔMXAN_0049* interact with each other. The bacterial colonies were removed gently at the point of interaction without disturbing the agar and transferred to sterile 1-mL Eppendorf tubes. Cells were broken by sonication on an ice slurry in the phosphate buffered saline (PBS) buffer (1 mM KH_2_PO_4_, 10 mM Na_2_HPO_4_, 137 mM NaCl, 2.7 mM KCl, pH 7.4), and centrifuged at 13,000 rpm for 30 min was performed to remove the unbroken cells and debris and to take the supernatant. The protocol for in-solution trypsin digestion was followed according to the method described by Leon et al. 2013 and Promega [24,25] with minor modifications. For LC-MS in solution digestion, a 10-μg sample was transferred to a 0.5 mL Eppendorf tube and mixed with 15 μL of denaturing buffer, followed by the addition of 3 μL dichlorodiphenyltrichloroethane (DDT) 1M and incubated for 2 h at 37 °C. The addition of 5 μL iodoacetamide (1M) was carried out, and the solution was incubated at room temperature in the dark for 1 h. For sample desalting, a Microcon YM-10 centrifugal filter unit was washed with 25 mM ammonium bicarbonate (500 μL), centrifuged at 14,000 rpm at 4 °C for 15 min, and the buffer was discarded. The sample (25 μL) was then transferred with the addition of 25 mM ammonium bicarbonate (470 μL), centrifuged at 14,000 rpm at 4 °C for 15 min, and the waste supernatant was discarded. HPLC-grade water (500 μL) was added in the centrifugal tube and centrifuged at 14,000 rpm at 4 °C for 40 min. Thereafter, the column was inverted in another collection tube, and the remnant was centrifuged at 1000× *g* for 3 min. The desalted samples were dried by vacuum drier Eppendorf Concentrator 5301. The samples were reconstituted with 19 uL of 25 mM ammonium bicarbonate for in-solution trypsin digestion and 9.5 μL of the sample to a 0.5 mL Eppendorf tube, and incubated with Trypsin Gold (cat#V5280, Promega™, Madison, WI, USA) mass spectrometry grade (1:25, *w/w*) at 37 °C overnight. Then, samples were purified through mZipTip C18 pipette tips (Millipore, Merck, Burlington, MA, USA) following the methodology reported previously by Zachara et al., 2011 and Breitkopf and Asara, 2012 [26,27].

### 2.5. Proteomics Analyses and Data Processing

Peptides prepared from in-solution digestion were analyzed by a nano-LC system (Eksigent Technologies, nano-LC-Ultra 2D plus, Woonsocket, RI, USA) coupled with a mass spectroscopy system (Thermo Scientific, LTQ Orbitrap velos pro ETD, Waltham, MA, USA). Three biological replicates were performed for each sample. Separation was accomplished on silica columns (75 μm × 25 cm) packed with Reprosil-Pur 120 C18-AQ (particle size 3 μm, DR. MAISCH GMBH, Entringen, Germany) with a mobile phase system of solvent A (2% acetonitrile (ACN) and 0.1% formic acid) and B (70% ACN and 0.1% formic acid) at a flow rate of 300 nL/min. The following stepping gradient program was applied: 0-5 min, 0% solvent B; 5–20 min, 0 to 10% B; 20–75 min, 10 to 32% B; 75–95 min, 32 to 50% B; 95–100 min, 50 to 100% B; 100–108 min, 100% B; 108–110 min, 100 to 0% B; and 110–120 min, 0% B. Eluted peptides were introduced in an MS system where the electrospray ionization (ESI) source ionized them at 2 kV, and the ionized gaseous peptide ions were sent to mass analyzer. The LTQ-Orbitrap Velos Pro ETD was run in data-dependent acquisition mode with Xcalibur 2.2.0 software (Thermo Scientific, USA). Full-scan MS spectra (from 350 to 1650 m/z) were detected in the Orbitrap with a resolution of 60,000 at 400 m/z. The 10 most intense precursor ions greater than the threshold of 5000 counts in the linear ion trap were selected for MS/MS fragmentation analyses at a normalized collision energy of 35%. In order to avoid repetitively selecting peptides, dynamic exclusion was used within 60 s. The raw files from LTQ-Orbitrap Velos Pro were imported into Proteome Discoverer software 1.4 (Thermo Scientific, USA) with the Sequest HT search engine against proteome sequence databases of DK1622 (UP000002402 in UniProt in RefSeq assembly) for data processing. The processing parameters were set as: (i) two maximum missed cleavage sites allowed in trypsin digestion; (ii) digested peptide length from 6 to 144; (iii) 10 ppm of precursor mass tolerance and 0.8 Da of fragment mass tolerance; (iv) oxidation of methionine in dynamic modification and carboxymethyl cysteine in static modification; (v) the false discovery rate (FDR) validation based on *q*-value < 0.01. Cut off for Sequest HT and for peptide spectrum matches (PSMs) was 0.05. The maximum ∆Cn was 0.05. The relative abundance of proteins was characterized by PSMs. Among the peptide filters or the grouping settings, peptide confidence level was selected as “high”.

### 2.6. T6SS-Knockout Sample Preparation

The deletion mutations were generated in the wild-type strain DK1622 using standard methods. Upstream and downstream regions of genes were amplified, ligated together, and cloned into the plasmid pBJ113. The deletion plasmids were electroporated into DK1622, where they integrated into the genome by homologous recombination. Strains were inoculated over CTT plates containing Km and then picked and screened on 1% D galactose CTT agar plates. The resulting deletion mutants were verified by PCR using primers flanking the deletion site. Different antibiotic resistance mutants of the deletion mutants and DK1622, tetracycline resistance, and kanamycin resistance were constructed. The Δ*MXAN_0049*-Tet was generated by electroporation of the plasmid pSWU30, which encodes tetracycline and integrates into the Mx8 attB site. DK-Km and Δ*MXAN_0049*-Km were generated using the plasmid pSWU19, which encodes kanamycin. attR primers with the sequences 5′-AAAAAAGCTTCCGGGCGGCCTTGCGGAATGAT-3′ and 5′ TCAGCGCTTCAGGTCCGGGACTGGGAC-3′ were used for integration [10].

### 2.7. Extraction of Compounds from Boundary

The extraction of compounds was carried out according to the methods described previously [28,29] with partial modifications. Briefly, fresh log phase cultures of DK1622 and mutant strains Δ*MXAN_0049* were point spotted as dots on CTT plates containing 1.5% agar with the help of sterile toothpicks and at the distance of 0.8 cm between two strains. They were incubated for 5 days at 30 °C. Then, the interacted zone (boundary) was sliced precisely under the stereomicroscope to avoid unwanted contaminants (beside the boundary colonies) and was transferred from plates to sterile tubes (50 mL). After that, sliced boundary zone was cut into small pieces, and 20 mL of 80% acetone (*v/v*) were added. The sample solution was shaken vigorously in an incubator shaker for 45–60 min at room temperature. Thereafter, the sample solution was centrifuged at 4000× *g* for 10 min, and the supernatant was carefully transferred into a clean tube by avoiding the solid constituents. The complete acetone evaporation was performed via open lid tubes in a shaker (until 25% of the solution was left). The water fraction was then acidified with trifluoroacetic acid (TFA) (0.1% (*v/v*), mixed with two volumes of ethyl acetate, shaken vigorously for 5 min at room temperature, and incubated overnight at −20 °C. The next day, the unfrozen (ethyl acetate) fraction that contained the active compounds was carefully transferred to a new flask (a 200–300 mL flask was more suitable) and dried under airflow. The dried extract was dissolved in 300–200 µL of 50% (*v/v*) methanol (HPLC gradient). Then, the solution was pipetted out, transferred into a 1.5 mL sterile tube, and centrifuged for 30 min at 13,000 rpm. Afterwards, the supernatant was filtered and, from the filtrate, a 50 µL volume was transferred to HPLC tubes for analysis. Experiments were carried out in three replicates.

### 2.8. Identification of Compounds Using High-Resolution HPLC-MS/MS System

HPLC-MS/MS was carried out by using a rapid separation liquid chromatography system (Dionex, UltiMate 3000, UHPLC, Fisher Scientific, Waltham, MA, USA) coupled to electrospray ionization quadrupole time-of-flight mass spectrometry (ESI-Q-TOF-MS) mass spectrometer (Bruker Daltonics, Impact HD, Bruker Daltonics Inc., Billerica, MA, USA). The separation was performed on a YMC-Pack pro C18 column (250 mm × 4.6 mm I.D., particle size 5 µm) with a mobile phase system in HPLC grade; (A) water (Milli-Q) + 0.1% formic acid (Merck KGaA, Darmstadt, Germany) and (B) acetonitrile (Fisher Scientific, Waltham, MA, USA) + 0.1% formic acid (Sigma) and the same gradient program previously reported [30]. DataAnalysis 4.2 software (Bruker Daltonics) was utilized to analyze the *m*/*z* values and the MS/MS fragment information.

### 2.9. Data Analysis

A Venn diagram was generated using an online tool, VENNY 2.1 (http://bioinfogp.cnb.csic.es/tools/venny/index.html). Pathway analysis and mapping were performed by using KOBAS v3.0 KEGG Orthology Based Annotation System (http://kobas.cbi.pku.edu.cn/). For protein–protein interaction, we used String (Protein–Protein Interaction Networks, V: 10.5) database (https://string-db.org/) and Protein Ontology (PRO; http://proconsortium.org).

### 2.10. Proteomic and Metabolomics Data Accession Numbers

For proteomics datasets, “The mass spectrometry proteomics data have been deposited to the ProteomeXchange Consortium via the PRIDE partner repository with the dataset identifier PXD012532”. To download the file: Username: reviewer50449@ebi.ac.uk and Password: DK5zKJqJ. For metabolomics datasets: 10.6084/m9.figshare.8197724.

## 3. Results

### 3.1. Colony Boundaries between Incompatible M. xanthus Strains under Microscope

**Figure 1:**
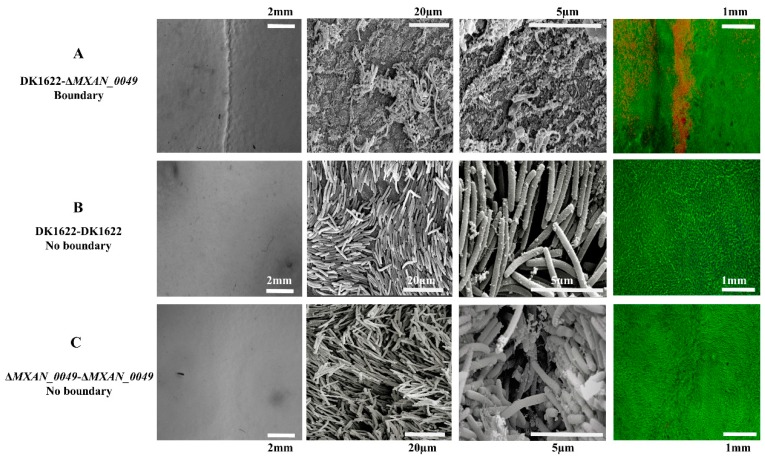
Status of boundary cells by LIVE/DEAD cell viability assay and SEM. At different magnifications, we observed the status of the cells at the point of interaction between DK1622 and *ΔMXAN_0049*, where a visible boundary appeared (**A**). At the point of interaction between same strains, DK1622-DK1622 and *ΔMXAN_0049*-*ΔMXAN_0049*, no boundary appeared (**B,C**). Cells within the boundary had irregular and damaged forms compared to the non-boundary, where cells were shown in a regular rod shape at 20 µm and 5 µm. LIVE/DEAD assay: the red line represents the damaged cells as the boundary at 1 mm (**A**), while the green color shows the active cells in the surrounding area (**B**,**C**).

We inoculated the *M. xanthus* DK1622 wild-type strain and the *MXAN_0049*-deletion mutant (Δ*MXAN_0049*) at the distance of 7 mm over CTT media (1.5% agar) in a pair-wise manner and incubated the plate for five days at 30 °C. Resultantly, a visible boundary was observed at the point of interaction between the DK1622 and the Δ*MXAN_0049* colonies (Figure 1A), while no boundary was formed in the case of the DK1622-DK1622 or the Δ*MXAN_0049*-Δ*MXAN_0049* colony interaction (Figure 1B,C). To check the condition of participated cells within the boundary, we utilized SEM for the high-end imaging. Irregular merged cell structures, vesicles, and damaged cell structures were observed within the DK1622-Δ*MXAN_0049* boundary (Figure 1A), while non-boundary sample cells of DK1622-DK1622 and Δ*MXAN_0049*-Δ*MXAN_0049* at the points of 20 µm and 5 µm were found to be normal and appropriately shaped (Figure 1B,C). Similarly, when the colonies were stained with the LIVE/DEAD^®^ *Bac*Light™ Bacterial Viability Kit, a clear line of red cells (cells with damaged/incomplete membranes) was visible in colony boundaries between DK1622 and Δ*MXAN_0049* at 1 mm (Figure 1A). While in the case of non-boundary as the control, most of the cells were alive and appeared in a green color at 1 mm (Figure 1B,C).

### 3.2. Proteomic Analysis of Colony Boundaries

**Figure 2:**
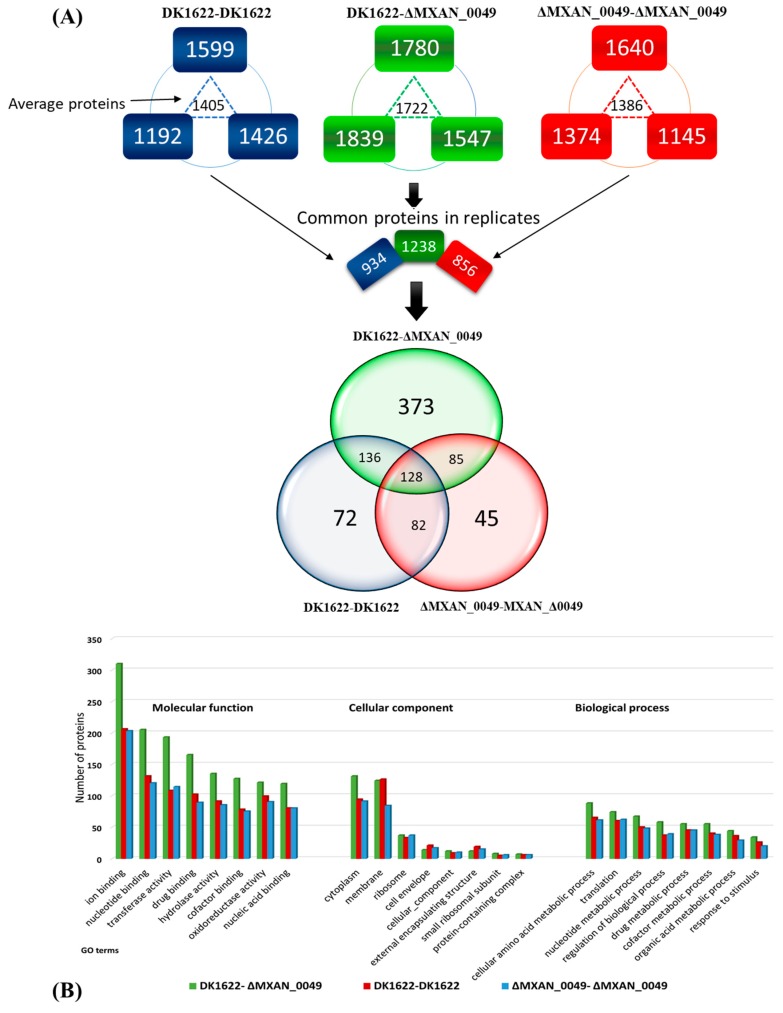
(**A**) Experimentally determined sub-proteomes of *M. xanthus 1622* by Venn diagram. The common proteins of all biological replicates were selected and compared with each other through the Venn diagram. There were 128 common proteins present in all three different combinations (boundary: Δ*MXAN_0049*-DK1622, non-boundary: DK1622-DK1622, and non-boundary: Δ*MXAN_0049-*Δ*MXAN_0049*) samples, while 373 unique proteins were observed when the boundary formed, and 72 and 45 unique proteins in the cases of both non-boundary samples (DK1622-DK1622 and Δ*MXAN_0049-*Δ*MXAN_0049*, respectively). (**B**) Ontological classification of the differentially regulated proteins within the boundary and the non-boundary and their comparison. The proteins were classified according to molecular function, cellular component, and biological process.

We utilized a proteomic approach to explore the alterations of the proteins during boundary formation. For that, we took the extracted protein samples from the interaction zones of DK1622-Δ*MXAN_0049*, Δ*MXAN_0049*-Δ*MXAN_0049*, and DK1622-DK1622. All the samples were processed through the in-solution digestion method with three biological replicates and were analyzed by a nano-LC-MS system (Eksigent Technologies, nano LC-Ultra 2D plus, Dublin, CA, USA). The generated results indicated the presence of participated proteins in DK1622-Δ*MXAN_0049* (1839, 1780, and 1547), in DK1622-DK1622 (1599, 1426, and 1192), and Δ*MXAN_0049*-Δ*MXAN_0049* (1640, 1374, and 1145) with the average values of 1722, 1405, and 1386 in DK1622-Δ*MXAN_0049*, DK1622-DK1622, and Δ*MXAN_0049*-Δ*MXAN_0049*, respectively. After that, we selected the common proteins in all three samples as 1238, 934, and 856 in DK1622-Δ*MXAN_0049*, DK1622-DK1622 and Δ*MXAN_0049*-Δ*MXAN_0049* along with 373, 72, and 45 unique proteins, respectively (Figure 2A); detailed data are listed in dataset S1. The ontologies of these proteins were analyzed through protein ontology (PRO). Gene ontology (GO slim) was categorized in the domains of cellular components, molecular functions, and biological processes (Figure 2B), and detailed data are presented in dataset S2.

In the molecular functions, annotation of the proteins in the DK1622-Δ*MXAN_0049* boundary showed that 310 proteins were in ion binding, while in the non-boundary samples, DK1622-DK1622 and Δ*MXAN_0049*-Δ*MXAN_0049*, there were 206 and 203, respectively. About 205 proteins, which played roles in nucleotide binding, appeared in the boundary, while in the non-boundary samples, DK1622-DK1622 and Δ*MXAN_0049*-Δ*MXAN_0049*, there were 131 and 120, respectively. In addition, the proteins related to transferase activity appeared more (239) in the boundary compared to both of the non-boundary samples. In the case of cellular components, 124 proteins of the membrane category were identified from the boundary, while 126 and 86 membrane proteins were categorized in the DK1622-DK1622 and the Δ*MXAN_0049-*Δ*MXAN_0049*. Similarly, in GO terms, the cell envelope (21, 17) and external encapsulating structure (19, 15) proteins were more represented in the non-boundary (DK1622-DK1622 and Δ*MXAN_0049-*Δ*MXAN_0049*) compared to the boundary (14 and 12). In the biological process domain, 88 proteins in the boundary represented the GO term cellular amino acid metabolic process compared to the non boundary samples (65, 61 in DK1622-DK1622 and Δ*MXAN_0049-*Δ*MXAN_0049* respectively). Similarly, 55 proteins represented the GO term drug metabolic process, while 45 proteins appeared in both non-boundary samples. The GO slim categorization showed that there were great differences between the proteins in the boundary and the non-boundary samples (Figure 2B).

**Figure 3:**
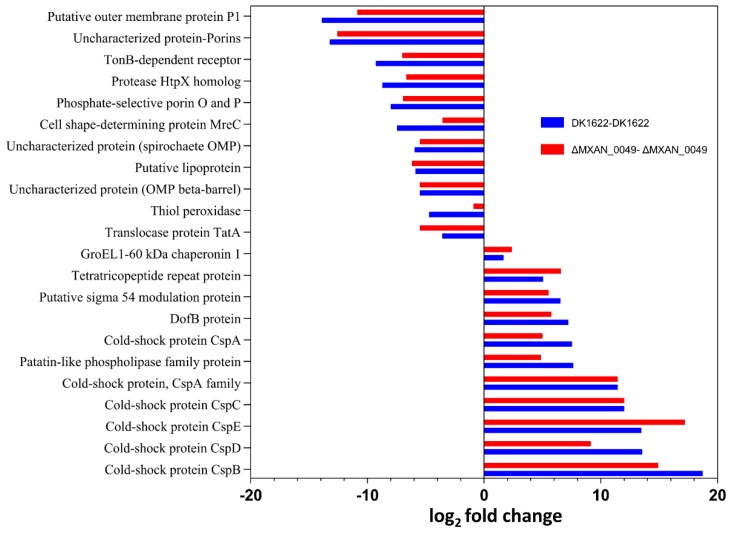
Different up- and down-regulated proteins when the boundary formed compared to the non-boundary samples based on coverage value. Cold shock proteins (Csps) were more than 10-fold up-regulated compared to both control samples. Other proteins such as patatin-like phosphate, Dof-B protein (DofB), tetratricopeptide repeat (TPR) and chaperonin GroEL1 were also significantly up-regulated in the boundary. Porins, putative outer membrane proteins P1, and TonB-dependent receptor were down-regulated significantly in the case of the boundary when compared with both control samples, DK1622-DK1622 and *ΔMXAN_0049*–*ΔMXAN_0049*.

For comparison of the proteins between the boundary and the non-boundary, we arranged the data on the basis of coverage and PSMs to find which proteins were present significantly and which proteins were down-regulated when the boundary formed compared to the non-boundary samples, DK1622-DK1622 and *ΔMXAN_0049*-*ΔMXAN_0049* (Figure 3, Table 1 and Table 2). We found that the most significant proteins were the stress-resistance proteins, such as GroEL1 and GroEL2, and cold shock proteins (CspB, CspC, CspD, and CspE) when the boundary formed. Others that were significantly higher in the boundary included DofB proteins, tetratricopeptide repeat proteins, and patatin-like phospholipase family proteins. In contrast, phosphate-selective porin O and P, TonB dependent, thiol peroxidase, cell shape-determining protein MreC, and OmpA family protein were down-regulated as the boundary formed.

#### 3.2.1. Significant Proteins and Their Co-Relation with Boundary Formation

Several previous studies reported that some Gram-negative bacterial species, such as *Legionella pneumophila* and *Rickettsia prowazekii*, have the capability to use patatin-like phospholipase as effector molecules to target host cellular membranes [31,32,33,34,35]. When the boundary formed, the patatin-like phospholipase (in *M. xanthus* DK1622, its MXAN_4295 with Uniprot accession, Q1D4F3) was up-regulated about 7.6-fold against DK1622-DK1622 and 4.6-fold against *ΔMXAN_0049*-*ΔMXAN_0049*. Similarly, tetratricopeptide repeat (TPR) proteins have been reported to be directly involved in the virulence-associated function in some bacterial pathogens (*Pseudomonas aeruginosa*, *Yersinia*, and *Francisella tularemia*) either by transferring the virulence factors into host cells or by connecting to host cells [36,37,38,39]. Overall, there are 154 proteins with the same homology as TPR within *M. xanthus* DK1622. When the boundary (DK1622–*ΔMXAN_0049*) formed, 41 of these TPR proteins were present, while 31 and 22 were present in the non-boundary cases of DK1622-DK1622 and *ΔMXAN_0049*-*ΔMXAN_0049*, respectively. Detailed data are listed in dataset S3.

Polyketide synthase (PKS) and non-ribosomal peptide synthetase (NRPS) are biosynthetic enzymes responsible for the production of many polyketides and non-ribosomal peptides, respectively [40]. Notably, 14 PKS/NRPs were present when the boundary formed, while only six and seven PKS/NRPS were present in DK1622-DK1622 and *ΔMXAN_0049*–*ΔMXAN_0049*, respectively. Detailed data are listed in dataset S3.

In the genome of *M. xanthus* DK1622, MreC is a shape-determining protein (MXAN_2645, Uniprot ID: Q1D90), which was reported to play a role in the formation of cell shape [41,42,43]. Interestingly, the MreC protein appeared in the non-boundary samples, while it was missing in the boundary samples, which probably explained the irregular and damaged cells over the boundary.

The porin domain proteins play a role in the survival of a cell, as they help cell by transporting nutrients or other substances into the cells and toxins and wastes out of the cells to prevent the accumulation of toxic substances [44]. There are 14 porin domain proteins in *M. xanthus* DK1622, nine of which were present in both the non-boundary samples, and these proteins were down-regulated when the boundary formed (Table 2 and Table S1). Similarly, TonB-dependent energy transduction is supposed to be an all-purpose system for the delivery of energy to the outer membrane for either the import or the efflux of important molecules [45]. There are 19 TonB-dependent receptors in *M. xanthus* DK1622, seven of which appeared in the boundary, while in the non-boundary samples of DK1622-DK1622 and *ΔMXAN_0049*-*ΔMXAN_0049*, 13 proteins in each were present (Table S2). Proteins such as TonB and porin proteins are responsible for the transportation and the delivery of waste, toxins, or other substances; they were either missing or down-regulated when the boundary formed. Consequently, toxic or other virulence substances that have to be removed or transferred from the cell stayed longer within the cells, causing partial death or damage of cells.

#### 3.2.2. Interaction of Cold Shock Proteins with RNA Degradation Pathway

CSPs are small proteins of about 70 amino acids, and they are highly conserved nucleic acid binding proteins, which regulate different gene expressions under conditions such as stress resistance, cell aggregation, and virulence related responses in bacteria (*Listeria monocytogenes* and *Enterococcus faecalis)* [46,47,48]. For example, CspA, CspB, and CspD within *L. monocytogenes* are vital in the regulation of stress resistance and virulence related functions [49], and the deletion of these genes not only decreases the capability of aggregation of cells but also the virulence [50]. In *M. xanthus* DK1622, there are six cold shock proteins (Table S3). Interestingly, when the boundary formed, all of them were present with a significantly higher coverage rate. In particular, CspB, CspC, and CspD were more than seven-fold higher compared to the control samples (non-boundary). It suggested that the stress level within the boundary was much higher than that in the non-boundary, and the CSPs probably affected the cell shape and the cell aggregation within the boundary. These cold shock proteins also belong to nucleic acid binding, oligonucleotide/oligosaccharide-binding fold family (OB-fold), which has virulence and stress-related function [51,52].

**Figure 4:**
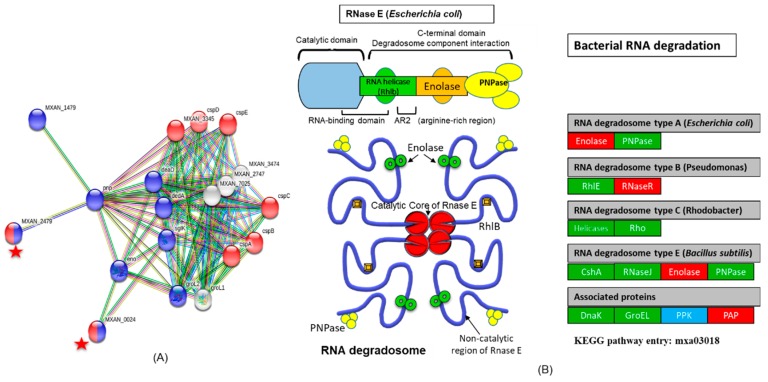
The protein–protein interaction network of cold shock proteins (CSPs) with RNA degradation pathway. (**A**) The 20 differentially expressed proteins were input into the STRING database (10.5 v) for protein–protein interaction (PPI) network analysis. We attained a PPI network of 20 nodes and 94 edges with PPI enrichment *p*-value < 1.0 × 10^−16^. The network nodes represent the proteins. Lines linking nodes with different colors represent the types of evidence used in prediction. Black line: co-expression evidence; green line: neighborhood evidence; purple line: experimental evidence; blue line: co-occurrence evidence; bright blue line: database evidence; red line: fusion evidence; and yellow line: text-mining evidence. CSPs have strong interactions with RNA degradation proteins. The interactions of these six CSPs proteins with transcription termination factor Rho and 3′-5′ exoribonuclease-RNaseR are represented with red stars. They together are part of nucleic acid binding, oligonucleotide/oligosaccharide-binding fold family (OB-fold). (**B**) The RNA degradation pathway modified from the original version taken from the KEGG pathway entry: mxa03018, https://www.genome.jp/kegg-bin/show_pathway?mxa03018 and Carpousis A.J, 2007 [53]. Here, we only present the protein present in *M. xanthus*. When the boundary formed, most of the proteins in RNA degradation were up-regulated, represented by green boxes, compared to the non- boundary, while red boxes represent the down-regulated proteins in this pathway. The blue box represents the protein that was not present in our data. In bacteria, endoribonuclease E, a key enzyme in RNA decay and processing, organizes a protein complex called degradosome. RNase R or E collaborates with PnPase (phosphate-dependent exoribonuclease polynucleotide phosphorylase) along with DEAD-box helicases and additional factors in the RNA-degrading complex. When the boundary appeared, these proteins up-regulated.

According to the STRING database (Protein–Protein Interaction Networks, V: 10.5), these CSPs have also strong interaction with RNA degradation proteins (Figure 4A). The 3′-5′ exoribonuclease-RNaseR (MXAN_0024, Uniprot ID: Q1DGB6) and transcription termination factor Rho (MXAN_2479, Uniprot ID: Q1D9H4) also belong to the OB-fold, and they are the main components of the RNA degradation pathway (Table S4). According to the KEGG database, the RNA degradation pathway in *M. xanthus* (pathway entry: mxa03018) has 14 proteins. All the main components of this pathway appeared when the boundary formed compared to the non-boundary samples. Out of the 14 proteins, nine were up-regulated and five were down-regulated when the boundary formed (Figure 4B). Detailed data are presented in Table S4 and dataset S3-KEGG pathways.

### 3.3. Co-Relationships of Boundary Formation with T6SS System

**Figure 5:**
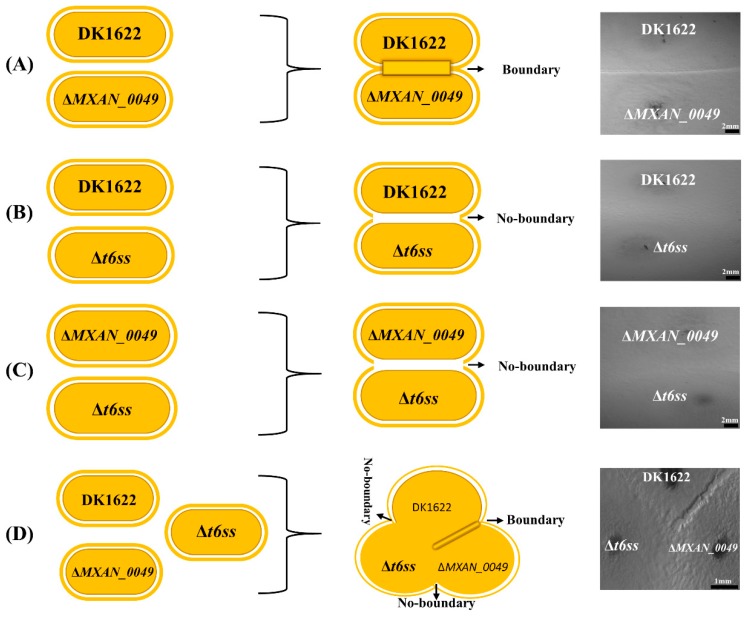
Knockout of T6SS gene cluster MXAN_4800-4813(Δ*t6ss*) for assessment of its role in the boundary formation. A visible boundary appeared at the point of interaction between DK1622 and Δ*MXAN_0049* (**A**). When DK1622 interacted with Δ*t6ss*, no boundary formed (**B**). Similarly, in the case of Δ*MXAN_0049* and Δ*t6ss* interaction, no boundary appeared (**C**). When all three strains, Δ*MXAN_0049*, Δ*t6ss*, and DK1622, interacted with each other on the same plate, only a visible boundary appeared in the case of DK1622 and Δ*MXAN_0049* at the point of interaction (**D**).

It is well known that bacteria transfer secreted effectors during competition through direct contact or from a distance approach [54,55]. T6SS is one of the effective mechanisms of Gram-negative bacterial secretion pathways [56,57]. *M. xanthus* DK1622 has a T6SS gene cluster (MXAN_4800–MXAN_4813) [58,59,60], and our previous studies suggested that T6SS components were involved in the colony-merger incompatibility between DK1622 and the Δ*MXAN_0049* mutant [10]. The proteomics data further provide evidence about the presence of essential components of T6SS during boundary formation, which gave us the possible hint of the T6SS role in boundary formation (Table S5). We deleted the entire T6SS gene cluster, MXAN_4800-4813 (Δ*t6ss*), from DK1622 and inoculated the Δ*t6ss* mutant with Δ*MXAN_0049* and DK1622 on the CTT (1.5%) agar plate. After five days of incubation, the T6SS knockout strain formed no boundary with either Δ*MXAN_0049* or DK1622, while DK1622 formed a normal strong boundary with Δ*MXAN_0049*. The disappearance of the boundary with Δ*MXAN_0049* after T6SS knockout from DK1622 strongly suggested the substantial role of the T6SS system in the boundary formation in *M. xanthus* DK1622 (Figure 5). The T6SS apparatus comprises a membrane-bound baseplate, an Hcp inner tube, a VipAB outer sheath, and a spike complex consisting of VgrG and PAAR proteins [6,57,58,59,60,61]. The VgrG protein, which is located at the tip of the T6SS apparatus, is responsible for delivering toxin proteins. There are two *vgrG* genes in *M. xanthus* DK1622, along with the second VgrG2 (MXAN_5573), which has 43% amino acid sequence similarity with VgrG (MXAN_4800) [62]. The proteomics data showed that the two kinds of VgrG proteins both appeared when the boundary formed. In the case of the control samples, both VgrG proteins were missing in the connection region between the Δ*MXAN_0049* and the Δ*MXAN_0049* colonies, and single VgrG (MXAN_4800) was present in the DK1622-DK1622 sample.

### 3.4. Identification of Chemical Compounds by HPLC-MS/MS

**Figure 6:**
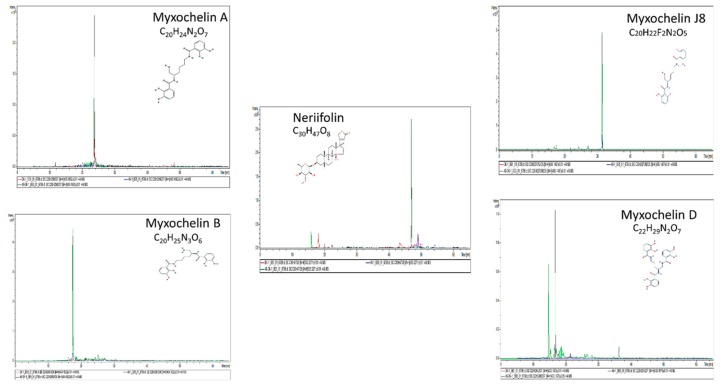
Production of different reported secondary metabolites during boundary formation. Different reported secondary metabolites were produced significantly when the boundary formed compared to the both controls (DK1622-DK1622 and Δ*MXAN_0049*-Δ*MXAN_0049*), which indicated the possible role of secondary metabolites as one of the factors affecting the cells and the formation of the boundary.

Among prokaryotes, myxobacteria are well known for their abilities to produce an extensive range of secondary metabolites [63,64,65,66]. Most of the reported secondary metabolites of *M. xanthus* DK1622 have a broad range of biological activities and are suggested to play different functions in survival and predation [67]. We further performed HPLC-MS/MS to detect chemical compounds present within the boundary (DK1622–Δ*MXAN_0049*) and in two controls (Δ*MXAN_0049*–*ΔMXAN_0049* and DK1622-DK1622) with non-boundary. The MS/MS analysis was carried out by Otof-Control software (Bruker Daltonics). The detected compounds were authenticated with the reported reference secondary metabolites produced by *M. xanthus* [30,65,68,69]. Several reported compounds, such as Myxochelin A [C_20_H_24_N_2_O_7,_ m/z 405.1656 (M + H)], Myxochelin B [C_20_H_25_N_3_O_6,_ *m*/*z* 404.1816 (M + H)]. Myxochelin D [C_22_H_29_N_2_O_7_, m/z 433.1969 (M + H)], Myxochelin J8 [C_20_H_22_F_2_N_2_O_5_, m/z 408.1584 (M + H)], and Neriifolin [C_30_H_47_O_8_, m/z 535.3265 (M + H)] (Figure 6, Table S6) were significantly present in the sample (DK1622–*ΔMXAN_0049*) compared to the control (DK1622-DK1622 and *ΔMXAN_0049*–*ΔMXAN_0049*). All these compounds were confirmed through Bruker’s SmartFormula and CompoundCrawler- 3.1.3, compounds, and the ppm value (˂5) and the mSigma value (˂20) were selected.

We also observed that some aromatic amino acids, such as phenylalanine, tyrosine, and tryptophan, appeared significantly higher when the boundary formed compared to the control (non-boundary samples). l-phenylalanine (C_9_H_11_NO_2_) with calculated m/z (M + H) 166.0863 was 110 and 54 folds higher in the boundary than it was in the DK1622-DK1622 and the Δ*MXAN_0049*-Δ*MXAN_0049* samples by area, respectively (Table 3, Figure S1A). Likewise, l-tyrosine (C_9_H_11_NO_3_) was also significantly high in the boundary. There were two peaks of l-tyrosine in the sample of DK1622-Δ*MXAN_0049* (boundary); the main peak was at the retention time (RT) of 6.8 min with a calculated m/z (M + H) of 182.0812, and the second peak was at 22 min (RT) with a calculated m/z (M + H) of 182.0812. The l-tyrosine in the boundary sample was 2.6 and 2.4 times more by area than the control samples DK1622-DK1622 and Δ*MXAN_0049*-Δ*MXAN_0049*, respectively (Table 3, Figure S1B). l-tryptophan (C_11_H_12_N_2_O_2_) appeared at RT 14.7 (min) with a calculated m/z (M + H) of 205.0972. The compound was 6.2 and 6.9 folds higher than it was in DK1622-DK1622 and *ΔMXAN_0049*-*ΔMXAN_0049* (non-boundary) by area, respectively (Table 3, Figure S1C).

**Figure 7:**
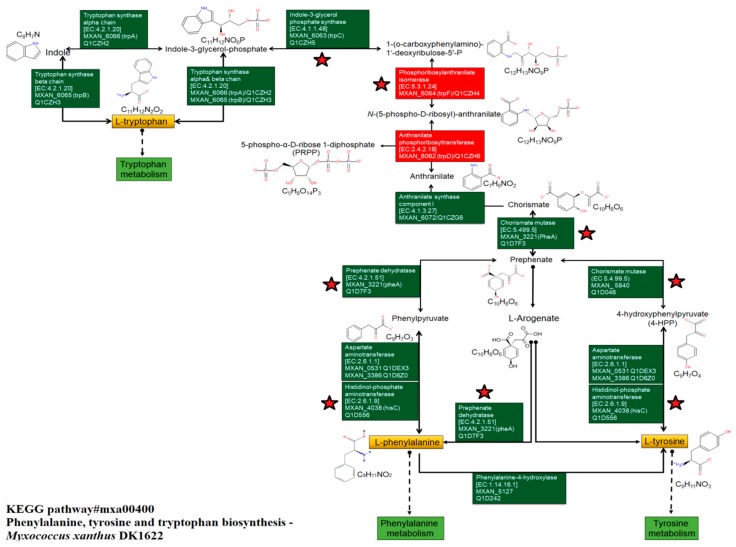
Phenylalanine, tyrosine, and tryptophan biosynthesis, KEGG Pathway ID: #mxa00400. When the boundary formed, the three aromatic amino acids, l-penylalanine, l-tyrosine, and l-tryptophan, biosynthesis also appeared. Chorismate is the common intermediary in the formation of all three aromatic amino acids. Green boxes represent the enzymes present when the boundary appeared, while red boxes represent enzymes missing in the boundary samples (Table S7) The red stars represent the enzymes missing in the non-boundary samples (DK166-DK1622). Pathway analysis was performed by using KOBAS v3.0 (KEGG Orthology Based Annotation System).

Chorismate plays the role of the common intermediary in the biosynthesis of the aromatic amino acids, l-phenylalanine, l-tyrosine, and l-tryptophan [70]. According to the KEGG pathway, chorismate also acts as the intermediary for the biosynthesis of phenylalanine, tyrosine, and tryptophan in *M. xanthus* (Figure 7). Notably, our proteomics data showed that all the essential enzymes that were required for the biosynthesis of the aromatic amino acids, l-phenylalanine, l-tyrosine, and l-tryptophan appeared when the boundary formed (Table S7). In the case of the non-boundary, several enzymes were missing for the formation of these aromatic amino acids. We suggested that the presence of the aromatic amino acids (l-phenylalanine, l-tyrosine, and l-tryptophan) was possibly the result of cells being poisoned, which confirmed one of the causes of dead/damaged cells within the boundary.

## 4. Discussion

Microbial sociology is directly influenced by neighbors’ synergistic or antagonistic behaviors, which are based on the ability of microbes to identify the self and exclude the non-self to form groups of related individuals [71,72]. Moreover, sometimes it is governed by the aggregation of cells and resultantly forms a fruiting body for further cell sporulation [1,73,74,75]. However, it comes over the production of competing cells; organisms utilize all their resources—from small and simple units to complex multicomponent mechanisms [76,77].

Notably, we focused on the *M. xanthus* DK1622 and the incompatible mutant, *ΔMXAN_0049*, which formed the morphologically visible demarcation line between swarms of approaching bacterial colonies (Figure 1A), which was referred to as the boundary formation [3,78]. We addressed this phenomenon with the antagonism prospective and referred to it as kin discrimination [79]. Cell-to-cell kin discrimination in bacteria often shows as colony-merger incompatibility and, until today, there were several studies about colony merge incompatibility in bacteria [2,3,4,5]. Kin-discriminatory demarcation lines are observed commonly in *Proteus mirabilis* [3,78], *Bacillus subtilis* (5), *M. xanthus* [80], and *Pseudomonas aeruginosa* [81]. High end SEM and confocal imaging of the *M. xanthus* DK1622-*ΔMXAN_0049* boundary profoundly declared the incompatibility between them. However, it was reported earlier that most genetically identical strains have no such demarcation line [12], which was positively displayed in the case of DK1622-DK1622 and *ΔMXAN_0049*-*ΔMXAN_0049* (Figure 5). However, recent studies have shown that mutations in several independent genetic loci lead to the incompatibility that was detected within the same sequence strains [1,21]. Previously, it was revealed that protein aggregation played a major factor in bacterial virulence and in defense mechanisms against the toxic threat of aggregates [82]. The current study of boundary formation with different approaches triggered our understanding regarding the direct or the indirect involvement of different genes, proteins, and pathways in boundary formation. Several genes and proteins were absent or down-regulated when the boundary formed. Based on both coverage and PSM values, we sorted out the proteins that were significantly high in their expression level. We found that when the boundary formed, many of the stress resistance proteins, such as the cold shock proteins, GroEL1 and GroEL2, expressed in high levels. More interestingly, 14 PKS/NRPs were found in the boundary proteins, while DK1622-DK1622 and Δ*MXAN_0049*-Δ*MXAN_0049* revealed six and seven, respectively. It was reported by several researchers that PKS/NRPs are the main skeletons for metabolic product biosynthesis that participate in major antibacterial activity [83,84].

Our proteomics data also showed that, when the boundary formed, the degradation of RNA also occurred, and the RNA degradation pathway was significantly active. Additionally, most of the key proteins were up-regulated compared to the non-boundary samples. In addition, a few other proteins, such as patatin-like phospholipase, tetratricopeptide repeat protein, and DofB protein, were significantly present when the boundary formed (more than two change folds). Some protein coverage significantly went down when the boundary formed, which might have led to the prominent effect over the cells within the boundary. Porins, lipoproteins, and the TonB-dependent receptor, which all play roles as passages of nutrients by transferring the signals, the waste products, and the proteins that help protect the cell against oxidative stress via detoxifying peroxides such as thiol-specific peroxidase, were down-regulated. The above study opened the mechanistic clues of bacterial microbiota of natural environments that have coexisted in distinct niches with diverse strains and have protection from resource competition or exploitation by other strains [12]. Moreover, the proteome study revealed the major causes of boundary formation between DK1622 and *ΔMXAN_0049*, which might have been due to the toxin not being expressed in the MXAN_0049 mutant. This was probably due to the mutation that made mRNA unstable.

*M. xanthus* is a well-known model strain and a significant source of different bioactive secondary metabolites [64,65]. The assessment of secondary metabolites revealed the presence of several metabolic compounds, such as Myxochelin A, Myxochelin B, Myxalamide D, Myxochelin J8, and Neriifolin, upon the boundary formation in contrast to the non-boundary. Imperative T6SS components existence revealed its role in the transportation of different components across the membrane; both VgrG proteins (MXAN_4800 and MXAN_5573) were found when the boundary formed. LIVE/DEAD viability assay and SEM displayed the presence of damaged/dead cells within the boundary, whereas active/alive cells were observed within both controls. We also found that, within the boundary, cell shape was changed, and they were not in a regular rod shape. It was reported previously that MreC played a role to form a rod shape in cells [40,41,42]. Our proteomics results showed that when the boundary formed, the MreC protein was absent, while it appeared in the control and cells found in regular shapes. Moreover, several pathways, such as phenylalanine, tyrosine, and tryptophan biosynthesis pathway were recovered during the boundary formation and were confirmed through metabolomic studies. The presence of phenylalanine, tyrosine, and tryptophan in the boundary indicated another possible factor of damaging/killing cells within the boundary. Overall, we can conclude that there are several pathways, proteins, metabolites, and toxins involved in the formation of the boundary, and the presented study elaborates upon the above factors via proteome and metabolome tools, which could be helpful for further research and to provide a better understanding of the complexity of boundary formation for further studies.

## Figures and Tables

**Table 1:** List of upregulated proteins in the boundary compared to the non-boundary samples.

Protein ID	Protein Description	Gene ID	Mean Value			Log_2_ (Fold Change)	
			DK1622–*ΔMXAN_0049* Boundary	*ΔMXAN_0049*-*ΔMXAN_0049* Non-Boundary	DK1622-DK1622 Non-Boundary	Boundary/*ΔMXAN_0049*	Boundary/DK1622
**Q1D3Y5**	GroEL1-60 kDa **chaperonin 1**	MXAN_4467	80.49	60.81	66.24	2.381892085	1.672802712
**Q1DEI3**	Cold-shock protein CspC	MXAN_0672	77.61	13.93	13.93	11.9978081	11.9978081
**Q1D1L2**	Cold-shock protein CspB	MXAN_5310	75.7567	7.07	1	14.90209495	18.72748517
**Q1D6F1**	Cold-shock protein CspD	MXAN_3582	64.1767	17.4167	6.96667	9.154352467	13.55123262
**Q1CZK1**	Cold-shock protein CspE	MXAN_6037	63.2333	16.86333	6.86333	17.19	13.48
**Q1D4F3**	Patatin-like phospholipase family protein	MXAN_4295	61.19	32.1267	20.9867	4.88	7.65
**Q1D4M7**	DofB protein	MXAN_4295	55.25	24.83	19.6867	5.77	7.21
**Q1DF46**	Putative sigma 54 modulation protein	MXAN_0457	37.83	15.91	13.21	5.54	6.53
**Q1D4P0**	Tetratricopeptide repeat protein	MXAN_4221	44.18	16.22	21.07	6.58	5.06
**Q1D730**	Cold-shock protein, CspA family	MXAN_3345	42.29	4.97	4.97	11.44	11.44
**Q1DBV4**	Cold-shock protein CspA	MXAN_1617	19.12	6.86333	2.94	4.99	7.54

**Table 2:** List of down regulated proteins in boundary.

Protein ID	Protein Description	Gene ID	Mean Value			Log_2_ (Fold Change)	
			DK1622–*ΔMXAN_0049* Boundary	*ΔMXAN_0049*-*ΔMXAN_0049* Non-Boundary	DK1622-DK1622 Non-Boundary	Boundary/*ΔMXAN_0049*	Boundary/DK1622
Q1CYN2	Putative lipoprotein	MXAN_6367	21.36	51.75	49.72	−6.20	−5.88
Q1D854	Sec-independent protein translocase protein TatA	MXAN_2960	19.57	44.20	34.06	−5.51	−3.60
Q1D031	Uncharacterized protein (Porin domain superfamily)	MXAN_5855	2.40	38.7	42.16	−12.59	−13.23
Q1CWS0	Putative outer membrane protein P1	MXAN_7040	1.51	27.70	42.57	−10.86	−13.89
Q1CZB5	Glyoxalase family protein	MXAN_6123	22.97	25.49	42.85	−0.66	−4.29
Q1CYA4	Thiol peroxidase	MXAN_6496	17.35	20.27	36.06	−0.91	−4.71
Q1CX48	TonB-dependent receptor	MXAN_6911	3.21	18.12	26.10	−7.03	−9.27
Q1DDT8	Uncharacterized protein (OMP beta-barrel)	MXAN_0924	3.47	14.22	14.22	−5.49	−5.49
Q1DEU3	Protease HtpX homolog	MXAN_0561	1.85667	14.21	20.4367	−6.66	−8.72
Q1D5R1	Uncharacterized protein (spirochaete OMP)	MXAN_3830	1.23	10.56	11.59	−5.51	−5.96
Q1DEU2	Phosphate-selective porin O and P	MXAN_0562	1	13.9333	16.9233	−6.93	−8.00

**Table 3:** Detection of phenylalanine, tyrosine, and tryptophan via HPLC-MS.

Compounds	Sample	Boundary/Non-Boundary	RT [min]	Calc. m/z [M + H]	Formula	Signal to-Noise (S/N)	Area	Intensity (I)	Difference with Boundary by Area (in Folds)
Phenylalanine	DK1622/*ΔMXAN_0049*	Boundary	11.5	166.0863	C_9_H_11_NO_2_	6118.5	2.02 × 10 ^8^	10,960,726	
	DK1622-DK1622	Non-boundary	11.6	166.0863	C_9_H_11_NO_2_	127.5	1,833,551	212,562	110.2998
	*ΔMXAN_0049*–*ΔMXAN_0049*	Non-boundary	11.6	166.0863	C_9_H_11_NO_2_	244.5	3,740,288	347,508	54.07075
Tyrosine	DK1622/*ΔMXAN_0049*	Boundary	6.8	182.0812	C_9_H_11_NO_3_	3017.4	53,175,768	5,498,402	
	DK1622-DK1622	Non-boundary	N.A	N.A	C_9_H_11_NO_3_	N.A	N.A	N.A	N.A
	*ΔMXAN_0049*–*ΔMXAN_0049*	Non-boundary	N.A	N.A	C_9_H_11_NO_3_	N.A	N.A	N.A	N.A
Tryptophan	DK1622/*ΔMXAN_0049*	Boundary	14.7	205.0972	C_11_H_12_N_2_O_2_	1761.1	18,269,390	2,708,408	
	DK1622-DK1622	Non-boundary	14.7	205.0972	C_11_H_12_N_2_O_2_	211.8	2,941,254	391,400	6.211429
	*ΔMXAN_0049*–*ΔMXAN_0049*	Non-boundary	14.8	205.0972	C_11_H_12_N_2_O_2_	207.5	263,8907	328,542	6.92309

*RT: retention time. *N.A: Not appeared within the sample.

## Supplementary Materials

The following are available online at https://www.mdpi.com/2073-4409/8/6/530/s1. Figure S1: Identification of l-phenylalanine (A), l-tyrosine (B), and l-tryptophan (C) in boundary extracts and comparison with non-boundary samples. Table S1: List of porin proteins within boundary. Table S2: TonB-dependent receptor. Table S3: List of cold shock proteins within boundary and non-boundary samples. Table S4: List of all components of RNA degradation pathway appeared within boundary and non-boundary. Table S5: List of boundary proteins in Type VI secretion system (T6SS). Table S6: Different secondary metabolites present within boundary as compared to non-boundary. Table S7: List of boundary proteins in phenylalanine, tyrosine, and tryptophan biosynthesis against non-boundary samples. Dataset S1: All common and unique proteins within boundary and non-boundary samples and processed data. Dataset S2: GO-slim. Dataset S3: List of TPR, PKS-NRPS, and selected KEGG pathways within boundary.
