# Supplementary material for: Omics Studies Revealed the Factors Involved in the Formation of Colony Boundary in Myxococcus xanthus
Source: Cells. 2019 Jun 3;8(6):530. doi: 10.3390/cells8060530 (PMC6627406; doi:10.3390/cells8060530)
Supplement: Supplementary file 1 [file cells-08-00530-s001.zip › Supplementary Tables S1-S7.pdf]

Omics Studies Revealed the Factors Involved in the Formation of Colony Boundary in *Myxococcus xanthus*

Mian Nabeel Anwar, Zhi Feng Li, Ya Gong, Raghvendra Pratap Singh and Yue-zhong Li *

State Key Laboratory of Microbial Technology, Institute of Microbial Technology, Shandong University, Qingdao 266237, P.R. China

Email addresses of the authors: miannabeelanwar@gmail.com lilab@sdu.edu.cn
* The corresponding author.

Supplementary tables (S1-S6)

Table S1: Porin proteins

Protein ID	Protein description	Gene ID	Mean value			Log2 (Fold change)	
			**DK1622-ΔMXAN_0049 Boundary**	**DK1622-DK1622 No-boundary 1**	**Δ*MXAN_0049*-Δ*MXAN_0049* No-boundary 2**	**Boundary/No boundary 1**	**Boundary/No boundary 2**
Q1D031	Uncharacterized protein	MXAN_5855	15.67333	42.16333	38.7	-6.42	-5.79
Q1DEU2	Phosphate-selective porin O and P	MXAN_0562	N.A	16.92333	17.94333	-8.00	-8.33
Q1D5R1	Uncharacterized protein	MXAN_3830	7.283333	13.79	10.56333	-2.94	-1.61
Q1D8U8	LPS-assembly protein LptD	MXAN_2708	0.833333	2.393333	0.836667	-0.72	0.001
Q1DDT8	Uncharacterized protein	MXAN_0924	3.476667	14.22	14.22	-5.49	-5.49
Q1D1X6	OmpA domain protein	MXAN_5194	12.18	19.54	13.30667	-2.58	-0.45
Q1CVR0	Uncharacterized protein	MXAN_7407	0	8.693333	4.99	-4.67	-2.58
Q1CZE8	Uncharacterized protein	MXAN_6090	9.696667	23.13	17.57667	-4.70	-3.056
Q1DFS2	Uncharacterized protein	MXAN_0219	5.153333	16.83333	10.69	-5.28	-2.92

- N.A = Not appeared in the sample

Table S2: TonB-dependent receptor

Protein ID	Protein Description	Gene ID	Mean value		
			DK1622-Δ*MXAN_0049* (Boundary)	DK1622-DK1622 (No-boundary)	Δ*MXAN_0049*-Δ*MXAN_0049* (No-boundary)
Q1DCB6	TonB-dependent receptor	MXAN_1450	59.1	57.56667	52.82667
Q1D365	TonB-dependent receptor	MXAN_4746	41.42333	40.93333	40.13333
Q1D2E5	TonB dependent receptor	MXAN_5023	15.92	24.77333	15.21
Q1CXB1	TonB-dependent receptor	MXAN_6845	8.256667	N.A	N.A
Q1D3P6	Putative tonB dependent receptor	MXAN_4559	6.32	10.28667	8.54
Q1DE39	TonB family protein	MXAN_0821	3.73	9.926667	9.02
Q1DEY6	Putative TonB-dependent receptor	MXAN_0518	3.026667	10.78667	7.933333
Q1CX48	TonB-dependent receptor	MXAN_6911	3.213333	26.10333	18.12333
Q1CY23	Putative TonB-dependent receptor	MXAN_6579	0.496667	10.91	6.476667
Q1DBN3	TonB family protein	MXAN_1688	N.A	6.33	2.3
Q1CVX9	TonB-dependent receptor	MXAN_7331	1.62	4.856667	1.99
Q1CZJ4	TonB-dependent receptor	MXAN_6044	1.856667	2.883333	2.003333
Q1DE06	TonB family protein	MXAN_0856	N.A	2.983333	2.103333
Q1DCQ0	Putative tonB dependent receptor	MXAN_1316	N.A	4.073333	1.936667

- N.A = Not appeared in the sample

Table S3: Cold shock proteins

Protein description	Gene ID	Uniprot ID	Mean value		
			**DK1622-Δ*MXAN_0049* (Boundary)**	**DK1622-DK1622 (No-boundary)**	**Δ*MXAN_0049*-Δ*MXAN_0049* (No-boundary)**
Q1DEI3	Cold-shock protein CspC	MXAN_0672	77.61	13.93	13.93
Q1CZK1	Cold-shock protein CspE	MXAN_6037	76.47	36.76	20.59
Q1D1L2	Cold-shock protein CspB	MXAN_5310	75.7567	7.07	N.A
Q1D6F1	Cold-shock protein CspD	MXAN_3582	64.1767	17.4167	6.96667
Q1D730	Cold-shock protein, CspA family	MXAN_3345	42.29	4.97	4.97
Q1DBV4	Cold-shock protein CspA	MXAN_1617	19.12	6.86333	2.94

- N.A = Not appeared in the sample

Table S4: RNA degradation pathway

Protein	Components in pathway	Unipot ID	Gene ID	Mean value		
				DK1622-Δ*MXAN_0049* (Boundary)	DK1622-DK1622 (No-boundary)	Δ*MXAN_0049*-Δ*MXAN_0049* (No-boundary)
Enolase	Eno	Q1D401	MXAN_4451	45.06333	59.33667	54.71
Polyribonucleotide nucleotidyltransferase	PnPase	Q1DAM1	MXAN_2073	40.16333	30.56667	36.61
3'-5' exoribonuclease	RNaseR	Q1DGB6	MXAN_0024	2.693333	0.633333	2.27
RNA helicase DeaD	Helicase	Q1D5I0	MXAN_3913	2.826667	N.A	3.156667
Transcription termination factor Rho	Rho	Q1D9H4	MXAN_2479	14.37	6.123333	3.413333
ATP-dependent helicase, DEAD/DEAH-box family	CshA	Q1D5I0	MXAN_3913	2.826667	N.A	3.156667
		Q1D015	MXAN_5871	11.75667	10.64333	9.28
Metallo-beta-lactamase family protein Ribonuclease J	RNaseJ	Q1DC87	MXAN_1479	4.09	N.A	0.793333
Putative ATP-dependent RNA helicase	RhlE	Q1D514	MXAN_4081	2.126667	N.A	N.A
Poly(A) polymerase I	pcnB	Q1D9A9	MXAN_2545	1.216667	6.263333	4.38
Heat shock protein 70 family protein	DnaK	Q1D8Q9	MXAN_2747	4.36	1.62	0
		Q1D6Q3	MXAN_3474	1.973333	N.A	0.803333
		P95334	MXAN_6671	10.76	2.306667	N.A
		Q1CWT5	MXAN_7025	7.893333	6.03	6.91
chaperonin GroEL	GroEL	Q1D3Y5	MXAN_4467	80.49667	60.81667	66.24
		Q1D2S1	MXAN_4895	83.42333	73.71	75.04667

- N.A = Not appeared in the sample

Table S5: Type 6 secretion system (T6SS)

Unipot ID	Gene	Protein	Components	Mean value		
				DK1622-Δ*MXAN_0049* (Boundary)	DK1622-DK1622 (No-boundary)	Δ*MXAN_0049*-Δ*MXAN_0049* (No-boundary)
Q1D0W1	MXAN_5573	Rhs element Vgr family protein	VgrG	3.88	N.A	N.A
Q1D312	MXAN_4800	Rhs element Vgr family protein	VgrG	2.076667	0.396667	0
Q1D310	MXAN_4802	hypothetical protein	Imp/Vas	3.59	N.A	1.466667
Q1D309	MXAN_4803	hypothetical protein	DotU	3.08	N.A	N.A
Q1D305	MXAN_4807	hypothetical protein	VipA	36.18	27.03	17.48
Q1D304	MXAN_4808	hypothetical protein	VipB	14.30667	13.49667	7.556667
Q1D303	MXAN_4809	hypothetical protein	Hcp	38.85333	30.87667	27.40333
Q1D2Z9	MXAN_4813	ATP-dependent chaperone protein ClpB	ATPase	3.993333	1.28	2.976667

- N.A = Not appeared in the sample

Table S6: Different secondary metabolites significantly present within boundary as compared to non-boundary.

Compound	Sample	RT [min]	Calc .m/z [M+H]	Formula	S/N (Signal)	Area	Intensity (I)	Difference with boundary by Area (in folds)
Myxochelin A	DK1622-*MXAN_0049*	23.8	405.1656	$C_{20}H_{24}N_2O_7$	317.9	2108247	244396	
	DK1622-DK1622	23.8	405.1656	$C_{20}H_{24}N_2O_7$	91	832396.1	75892	2.53
	Δ*MXAN_0049*-Δ*MXAN_0049*	23.8	405.1656	$C_{20}H_{24}N_2O_7$	17.1	131564	14720	16.02
Myxochelin B	DK1622-*MXAN_0049*	17.6	404.1816	$C_{20}H_{25}N_3O_6$	639.3	4192147	445198	
	DK1622-DK1622	17.6	404.1816	$C_{20}H_{25}N_3O_6$	96.7	855553	100828	4.89
	Δ*MXAN_0049*-Δ*MXAN_0049*	17.5	404.1816	$C_{20}H_{25}N_3O_6$	34.2	50265	22660	83.40
Myxochelin D	DK1622-*MXAN_0049*	16.8	433.1969	$C_{22}H_{29}N_2O_7$	1556.4	7247738	1027842	
	DK1622-DK1622	16.8	433.1969	$C_{22}H_{29}N_2O_7$	6.8	145751	14358	49.72
	Δ*MXAN_0049*-Δ*MXAN_0049*	16.9	433.1969	$C_{22}H_{29}N_2O_7$	3.5	18963.1	2598	382.20
Myxochelin J8	DK1622-*MXAN_0049*	31.5	408.1584	$C_{20}H_{22}F_2N_2O_5$	1082.6	4607838	484542	
	DK1622-DK1622	31.5	408.1584	$C_{20}H_{22}F_2N_2O_5$	140.6	417100.6	56648	11.04
	Δ*MXAN_0049*-Δ*MXAN_0049*	N.A	408.1584	$C_{20}H_{22}F_2N_2O_5$	170.8	472527.6	54878	9.75
Neriifolin	DK1622-*MXAN_0049*	47	535.3265	$C_{30}H_{47}O_8$	539.6	2158950	270494	
	DK1622-DK1622	47	535.3265	$C_{30}H_{47}O_8$	3.6	67637.5	8054	33.58
	Δ*MXAN_0049*-Δ*MXAN_0049*	47	535.3265	$C_{30}H_{47}O_8$	4.9	75890.9	5124	52.78

Table S7 : Proteins in Phenylalanine, tyrosine and tryptophan biosynthesis (KEGG Pathway ID #mxa00400)

Protein (Uniprot ID)	Protein Name /Enzyme ID	Gene ID	DK1622-Δ*MXAN_0049* (Boundary)	DK1622-DK1622 (No-boundary)	Δ*MXAN_0049*-Δ*MXAN_0049* (No-boundary)
			Mean value (coverage)		
**Phenylalanine biosynthesis**					
Q1D7F3	Chorismate mutase/prephenate dehydratase; (EC=5.4.99.5)	MXAN_3221	1.23	0	0
Q1DEX3	Aminotransferase, class I [EC:2.6.1.1]	MXAN_0531	24.34667	21.81333	27.03667
Q1D6Z0	Aminotransferase; [EC=2.6.1.9]	MXAN_3386	36.95333	32.57667	28.19667
Q1D556	Histidinol-phosphate aminotransferase [EC=2.6.1.9]	MXAN_4038	7.99	1.193333	0.826667
**Tyrosine biosynthesis**					
Q1D7F3	Chorismate mutase/prephenate dehydratase; (EC=5.4.99.5)	MXAN_3221	1.23	0	0
Q1D242	Aromatic amino acid hydroxylase, biopterin-dependent [EC:1.14.16.1]	MXAN_5127	4.063333	1.016667	3.746667
Q1D046	prephenate dehydrogenase [EC:1.3.1.12]	MXAN_5840	0	0	0
Q1DEX3	Aminotransferase, class I [EC=2.6.1.9]	MXAN_0531	24.34667	21.81333	27.03667
Q1D6Z0	Aminotransferase; [EC=2.6.1.9]	MXAN_3386	36.95333	32.57667	28.19667
Q1D556	Histidinol-phosphate aminotransferase; (EC=2.6.1.9;	MXAN_4038	7.99	1.193333	0.826667
**Tryptophan biosynthesis**					
Q1D7F3	Chorismate mutase/prephenate dehydratase; (EC=5.4.99.5)	MXAN_3221	1.23	0	0
Q1CZG6	Putative anthranilate synthase, component I [EC:4.1.3.27)	MXAN_6072	5.7	2.213333	4.763333
Q1CZH6	Anthranilate phosphoribosyltransferase (EC:2.4.2.18)	MXAN_6062 (trpD)	0	2.76	0
Q1CZH4	Phosphoribosylanthranilate isomerase (EC:5.3.1.24)	MXAN_6064 (trpF)	0	0	0
Q1CZH5	Indole-3-glycerol phosphate synthase (EC=4.1.1.48)	MXAN_6063 (trpC)	10.68667	0	0
Q1CZH2	Tryptophan synthase alpha chain (EC=4.2.1.20)	MXAN_6066 (trpA)	2.533333	1.266667	0
Q1CZH3	Tryptophan synthase beta chain (EC=4.2.1.20)	MXAN_6065 (trpB)	18.28667	13.97	9.976667
