# Supplementary figures and images for: Omics Studies Revealed the Factors Involved in the Formation of Colony Boundary in Myxococcus xanthus
Source: Cells. 2019 Jun 3;8(6):530. doi: 10.3390/cells8060530 (PMC6627406; doi:10.3390/cells8060530)

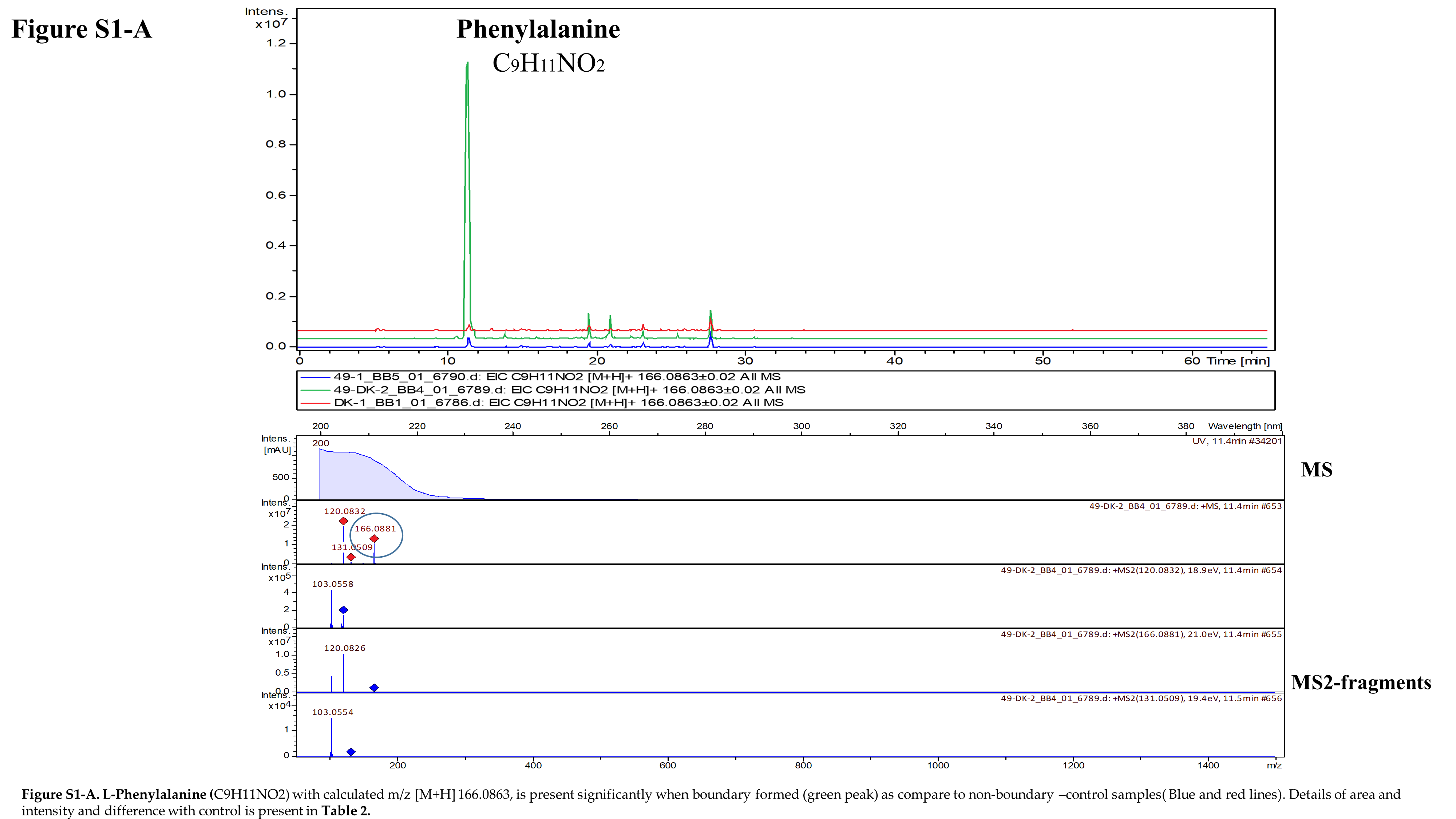

Supplement: Supplementary file 1 [file cells-08-00530-s001.zip › Supplementary Figure S1(A-C)/Figure S1-A.TIF]

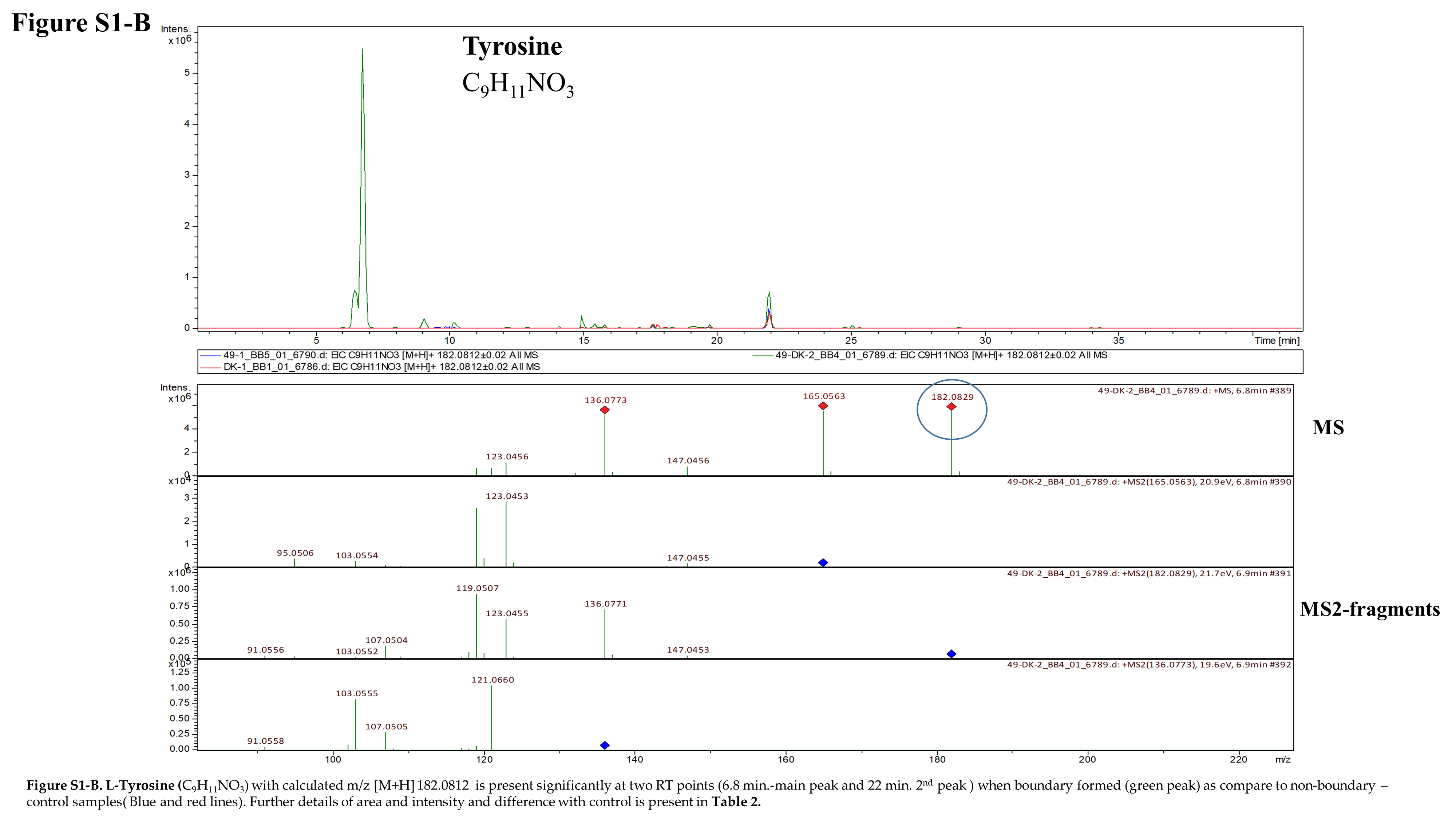

Supplement: Supplementary file 1 [file cells-08-00530-s001.zip › Supplementary Figure S1(A-C)/Figure S1-B.TIF]

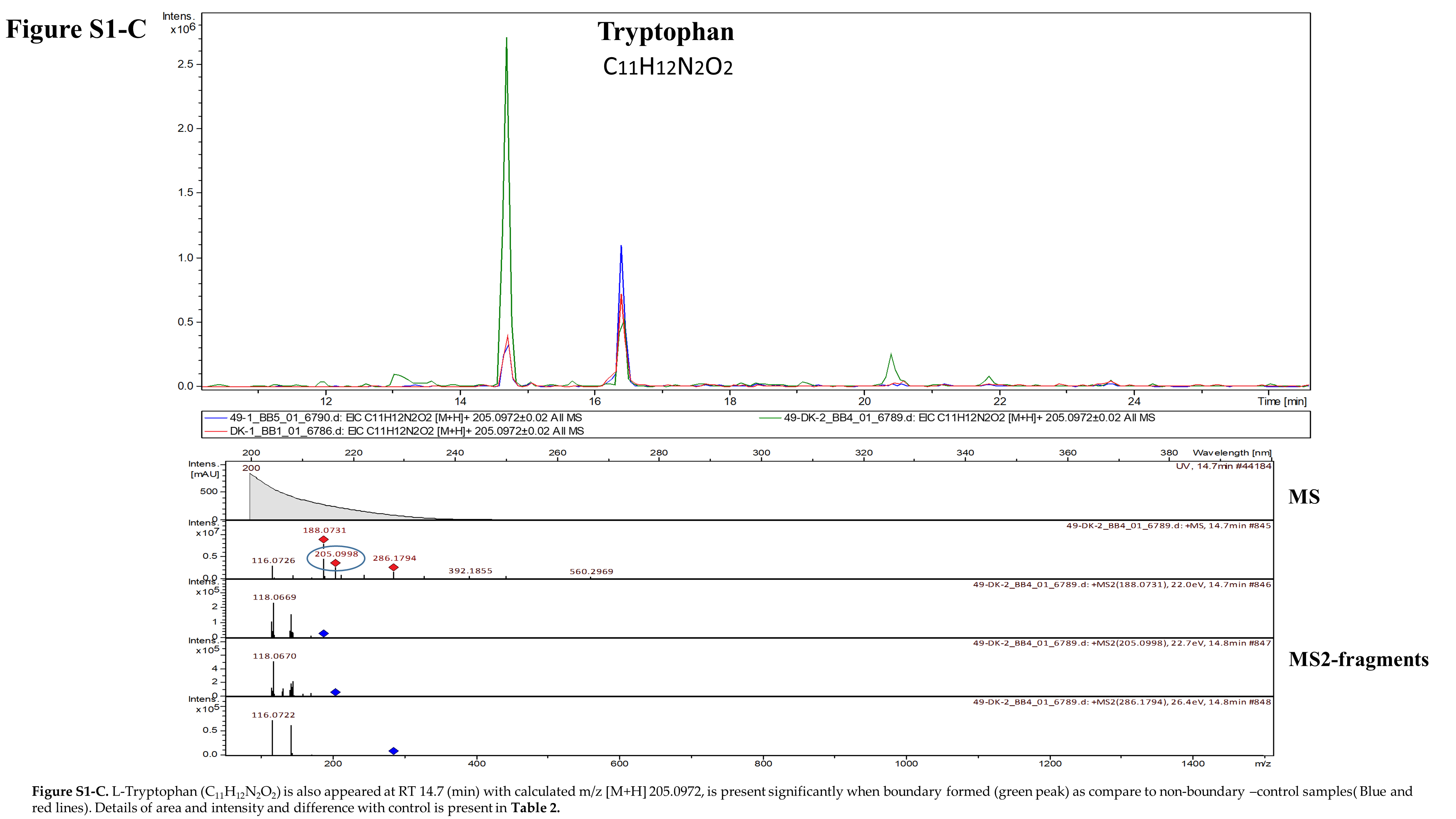

Supplement: Supplementary file 1 [file cells-08-00530-s001.zip › Supplementary Figure S1(A-C)/Figure S1-C.TIF]
